# Domain III of *Bacillus thuringiensis* Cry1Ie Toxin Plays an Important Role in Binding to Peritrophic Membrane of Asian Corn Borer

**DOI:** 10.1371/journal.pone.0136430

**Published:** 2015-08-21

**Authors:** Dongmei Feng, Zhen Chen, Zhiwen Wang, Chunlu Zhang, Kanglai He, Shuyuan Guo

**Affiliations:** 1 School of Life Science, Beijing Institute of Technology, Beijing, China; 2 Institute of Plant Protection, Chinese Academy of Agricultural Sciences, Beijing, China; Instituto de Biotecnología, Universidad Nacional Autónoma de México, MEXICO

## Abstract

The insecticidal IE648 toxin is a truncated Cry1Ie protein with increased toxicity against Asian corn borer (ACB). Cry toxins are pore-forming toxins that disrupt insect midgut cells to kill the larvae. However, the peritrophic membrane (PM) is an important barrier that Cry toxins must cross before binding to midgut cells. Previously, it was shown that Cry toxins are able to bind and accumulate in the PM of several lepidopteran insects. Binding of IE648 toxin to PM of ACB was previously reported and the goal of the current work was the identification of the binding region between Cry1Ie and the PM of ACB. Homologous competition binding assays showed that this interaction was specific. Heterologous competition binding assays performed with different fragments corresponding to domain I, domain II and domain III allowed us to identify that domain III participates in the interaction of IE648 with the PM. Specifically, peptide D3-L8 (corresponding to Cry1Ie toxin residues 607 to 616), located in an exposed loop region of domain III is probably involved in this interaction. Ligand blot assays show that IE648 interact with chitin and PM proteins with sizes of 30, 32 and 80 kDa. The fact that domain III interacts with proteins of similar molecular masses supports that this region of the toxin might be involved in PM interaction. These data provide for the first time the identification of domain III as a putative binding region between PM and 3D-Cry toxin.

## Introduction

Crop production has been compromised by insect pests since the beginning of agriculture. Insect control is now mainly achieved by chemical insecticides. However, this practice has resulted in serious negative effects on the environment because of their toxicity to non-target animals and humans [1]. The bacterium *Bacillus thuringiensis* (Bt) is the most successful insect pathogen used for insect control, constituting 2% of the total insecticidal market [2]. Bt Cry δ-endotoxins are highly specific insecticidal proteins synthesized during the sporulation phase of the bacterium. These toxins act at the midgut epithelial surface of the larvae of many species. However, insects can develop resistance to Cry toxins, which threatens the development and use of Bt toxins [3]. Insect resistance to Cry toxin action has been developed by different mechanisms, the most important correlated with altered binding to different receptors, which are located in the brush border membrane of the larval midgut [3–7].

The Bt *cry1I* genes [8] (formerly named *cryV* before Bt nomenclature standardization [9]) are silent in Bt strains but can be over-expressed in *Escherichia coli* cells. Cry1I proteins, which have a molecular mass of 81 kDa, are toxic to various lepidopteran larvae such as *Ostrinia nubilalis* and *Epiphyas postvittana* [8,10–12]. Cry1I proteins are also toxic against coleopteran insects, such as *Leptinotarsa decemlineata* [8] and *Tenebrio molitor* [10]; and one dipteran insect, *Culex pervigilans* [10]. The truncated Cry1Ie protein IE648, which corresponds to the first 648 amino acids of Cry1Ie, was shown to have improved insecticidal activity (LC_50_ to *Plutella xylostella* 1112.43 ng/mL) compared to the full-length Cry1Ie protein (LC_50_ to *Plutella xylostella* 1685.88 ng/mL) [13]. IE648 does not compete for Cry1Ac binding sites in the brush border membrane vesicles (BBMV) of the Asian corn borer (ACB), implying that IE648 binds to different receptors and thus may be a good candidate as part of a multiple-toxin strategy for the control of the ACB insect pest [14].

The peritrophic membrane (PM) of insects acts as the major barrier through which Cry toxins must cross before binding to receptors located in the brush border membrane. PM is an insoluble acellular matrix consisting of chitin fibrils and tightly associated proteins called peritrophins. This matrix separates the ingested food in the gut from the gut epithelium [15]. Chitin, the second most abundant polysaccharide on earth, is composed of β-1,4 linked N-acetylglucosamine moieties (GlcNAc) [16]. The peritrophins consist of at least two classes of glyconjugates: glycoproteins endowed with O-linked and N-linked oligosaccharides and proteoglycans with chains of glycosaminoglycans [17]. These glyconjugates endow the PM with multi-affinity binding capabilities to entrap microbial pathogens and toxic macromolecules, thus protecting the insect gut epithelial cells from injury and infection. Because of its semi-permeable characteristic, the PM can also enhance the digestive efficiency [18].

Previously, it was shown that Cry toxins are able to bind to and accumulate in the PM of various lepidopteran insects [6,17,19–23]. Valaitis and Podgwaite localized Cry1A toxin-binding sites in the PM and BBMV from Douglas-fir tussock moth (DFTM) larval gut and they provided strong evidence that glyconjugates endowed with O-glycans are indispensable for their interaction [17]. Furthermore, Cry toxins are able to pass through the PM to bind to their receptors located at the brush border membrane [24]. The rate at which the different Cry toxins can traverse the PM may differ, thereby affecting the resulting insecticidal activity. For example, it has been reported that the rate of penetration of the *Bombyx mori* PM is higher for Cry1Aa than for Cry1Ac, which correlates with the higher sensitivity of the *B*. *mori* larvae to Cry1Aa than to Cry1Ac [6]. To improve the toxicity of Bt toxins, several agents aimed at destroying PM, including *Cydia pomonella* granulovirus GP37 and chitinase have been investigated. These agents resulted in a significant enhancement of the lethality of Bt toxins in various types of larvae [23,25–29].

IE648, is a 3D-Cry toxin, that shows important insecticidal effect against ACB [13]. It was previously shown that IE648 interacts with the PM of ACB [14]. In this study, we focused to further analyze this interaction and define the region of Cry1Ie that is important for PM binding. These data provide useful information for understanding the mechanism of interaction between PM and the 3D-Cry toxin that could help to elucidate its role on the toxicity of Bt Cry proteins.

## Materials and Methods

### 2.1. Materials

In this work we used a truncated version of *cry1Ie* gene, containing the first 648 amino acid residues of Cry1Ie, cloned in vector pET-21b (Novagen) and named pET1Ie-648 [30]. This recombinant plasmid was expressed in *E*. *coli* BL21 (DE3), from Novagen. The full length of *cry1Ie* gene [8], in pET1Ie was provided by Institute of Plant Protection, Chinese Academy of Agricultural Sciences and only used to amplify the individual domains of this protein.

Ni-Agarose was purchased from QIAGEN. All other reagents were local products of analytical grade. All peptides were synthesized by SBS Co. Ltd in Beijing, China. Chitin (poly N-acetyl-1, 4-β-D-glucopyranosamine) from shrimp shells is from Sigma. Inclusion Body Solubilization and Refolding Kit from Tiandz, Inc Co. Ltd in Beijing, China. Biotin Tag Micro Biotinylation Kit from Sigma-Aldrich Co. LLC, United States.

### 2.2. Cloning of *cry1Ie* fragments encoding Cry1Ie protein domains I, II and III

Primer sequences for amplification of three domains of *cry1Ie* are shown in Table 1. pET1Ie was used as template. The PCR conditions were as follows: denaturation at 94°C for 30 s, annealing at 52°C for 45 s, and extension at 72°C for 1 min for 30 cycles. The obtained PCR products were digested with *Bam*HI and *Sal*I and inserted into vector pET21b. Transformed *E*. *coli* JM110 were selected with 100 μg/ml ampicillin. Gene fragments encoding different domains of Cry1Ie were confirmed by DNA sequencing. *E*. *coli* BL21 (DE3) cells were transformed with each recombinant plasmid for expression.

**Table 1:** Primer sequences for amplification of the three domains of *cry1Ie*.

Domain I	5′-CGC GGA TCC GAT GAA ACT AAA GAA TCC AG-3′
	5′-ACG CGT CGA CTG TAT CAT AGC TTG GGA AT-3
Domain II	5′-CGC GGA TCC GCT GGT ATA TCC AAT TA-3′
	5′-ACG CGT CGA CAT CTG CAC TAC GAT GTG-3′
Domain III	5′-CGC GGA TCC GAC AAT TGA GCC AAA TAG-3′
	5′-ACG CGT CGA CCA TGT TAC GCT CAA TAT GG-3′

### 2.3. Expression and purification of Cry1Ie IE648 protein and the three domains of Cry1Ie protein

IE648 protein was purified as previously described [14]. The different domains of Cry1Ie were expressed in *E*.*coli* BL21 (DE3) containing the corresponding recombinant plasmid, induced by 0.4 mM IPTG at 20°C for 20 h. The three domains were all expressed mostly as inclusions. Purification of domain I was described in a previous report [30]. The inclusion of domain III was dissolved by 50 mM Na_2_CO_3_, pH 10.2 as same as domain I. Domain II was dissolved by using the solubilization solutions of Inclusion Body Solubilization and Refolding Kit. After dissolved they were both purified by Ni-NTA (QIAGEN) as they have His-tags in their N-terminal, just like domain I, but different elution requirements. Domain II was eluted with 50 mM Na_2_CO_3_, pH 10.2. Domain III was eluted by 50 mM imidazole (50 mM Na_2_CO_3_, pH 10.2). Each one of the there domains present in Cry1Ie toxin has several lysine residues (34 in domain I, 20 in domain II and 11 in domain III), thus the purified domains were efficiently labeled with biotin using the BAC-Sulfo-NHS of Biotin Tag Micro Biotinylation Kit following the procedure of the kit and biotin labeling was verified through dot blotting.

### 2.4. Preparation of PM and BBMV

BBMV were prepared from midgut tissue isolated from fourth-instar ACB as described by Jurat-Fuentes *et al*. [31]. Aminopeptidase activity was used as a marker of enrichment for brush border membranes. BBMV were stored as aliquots at -80°C.

The entire larval PM was removed from midgut tissue and washed twice in cold phosphate buffered saline (PBS) before it was homogenized in cold PBS with protease inhibitors on ice using a Dounce homogenizer for 30 strokes [23]. The protein was quantified by the Bradford assay [32] using bovine serum albumin (BSA) as the standard and stored as aliquots at -80°C.

### 2.5. Competitive binding assay

Twenty micrograms of BBMV or PM proteins of ACB were incubated with 10 nM biotinylated IE648 in the presence or absence of a 50- to 1000-fold excess of unlabeled toxin in binding buffer (PBS, 0.1% w/v bovine serum albumin, 0.1% v/v Tween 20 at pH 7.6) for 1 h. The unbound toxin was removed by centrifugation for 10 min at 14,000 ×g. The BBMV or PM pellets were suspended in 100 μl of binding buffer and washed twice with the same buffer. Finally, the BBMV or PM were suspended in 20 μl of PBS, pH 7.6, and an equal volume of 2X-Laemmli sample loading buffer (0.125 M Tris—HCl, pH 6.8, 4% sodium dodecyl sulfate, 20% glycerol, 10% 2-mercaptoethanol, and 0.01% bromophenol blue) was added. Samples were boiled 5 min, loaded onto SDS—PAGE and electrotransferred to PVDF membranes [33]. The biotinylated protein that remained bound to the vesicles was revealed with streptavidin-peroxidase conjugate (1:10,000 dilution) for 1 h and visualized using the ECL Western Blotting Substrate (Pierce) according to the manufacturer’s directions.

### 2.6. Binding to chitin

Forty micrograms of insoluble chitin was incubated with 10 nM biotinylated protein (IE648, domain III or BSA) in binding buffer (PBS, 0.1% w/v bovine serum albumin, 0.1% v/v Tween 20 at pH 7.6) for 1 h. The unbound toxin was removed by centrifugation for 10 min at 14,000 ×g. The pellet was suspended in 100 μl of binding buffer and washed twice with the same buffer. Finally, the pellet was suspended in 20 μl of PBS, pH 7.6, and an equal volume of 2X -Laemmli sample loading buffer was added. Samples were boiled 5 min, loaded onto SDS—PAGE and electrotransferred to PVDF membranes [33]. The biotinylated protein that remained bound to the chitin was identified by incubation with Streptavidin-peroxidase conjugate (1:10,000 dilution) for 1 h and visualized using the ECL Western Blotting Substrate (Pierce) according to the manufacturer’s directions. Insoluble chitin with the same treatment and three biotinylated proteins alone were included as control.

### 2.7. Ligand blotting assay

PM of 20 ACB larvae were separated into four classes of components. S1 is the suspension obtained after PM dissolving in 1% Triton X-100 for 1 h at room temperature. Then grind the PM suspending in 1% Triton X-100. S2 is the homogenate obtained after grinding. S3 is the supernatant obtained after the centrifugation of the homogenate and S4 is the pellet obtained after the centrifugation. After SDS-PAGE of the four PM constituents (S1–S4), the PM proteins in the gel were electrotransferred onto PVDF membranes. The membranes were blocked by incubation with 5% BSA in PBS, pH 7.6 for 1 h, followed by washing five times for 10 min each with washing buffer (PBS containing 0.05% Tween 20). Then the membranes were incubated with 10 nM biotinylated toxin or domains in PBS for 1 h. Unbound toxin or domains were removed by washing with washing buffer five times for 10 min each. The biotinylated protein that remained bound to the membranes was revealed by incubation with streptavidin-peroxidase conjugate (1:10,000 dilution) for 1 h and visualized using the ECL Western Blotting Substrate (Pierce) according to the manufacturer’s directions.

## Results

### 3.1. Expression and purification of IE648 and the three domains of Cry1Ie protein

To determine which region of Cry1Ie is involved in binding to PM, the three structural domains were defined by multiple sequence alignments with other Cry toxins and a model of the three dimensional structure of Cry1Ie toxin was constructed. According to the multiple sequence alignment of the Cry toxins (Cry1Ia, Cry1B, Cry1Aa, Cry8E and others) and the structural predictions generated by Swiss Modeling, different domains of Cry1Ie toxin were designated as follows: domain I includes amino acids M1-T280, domain II includes amino acids V282-D497, and domain III includes amino acids T501-M719.

**Fig 1:**
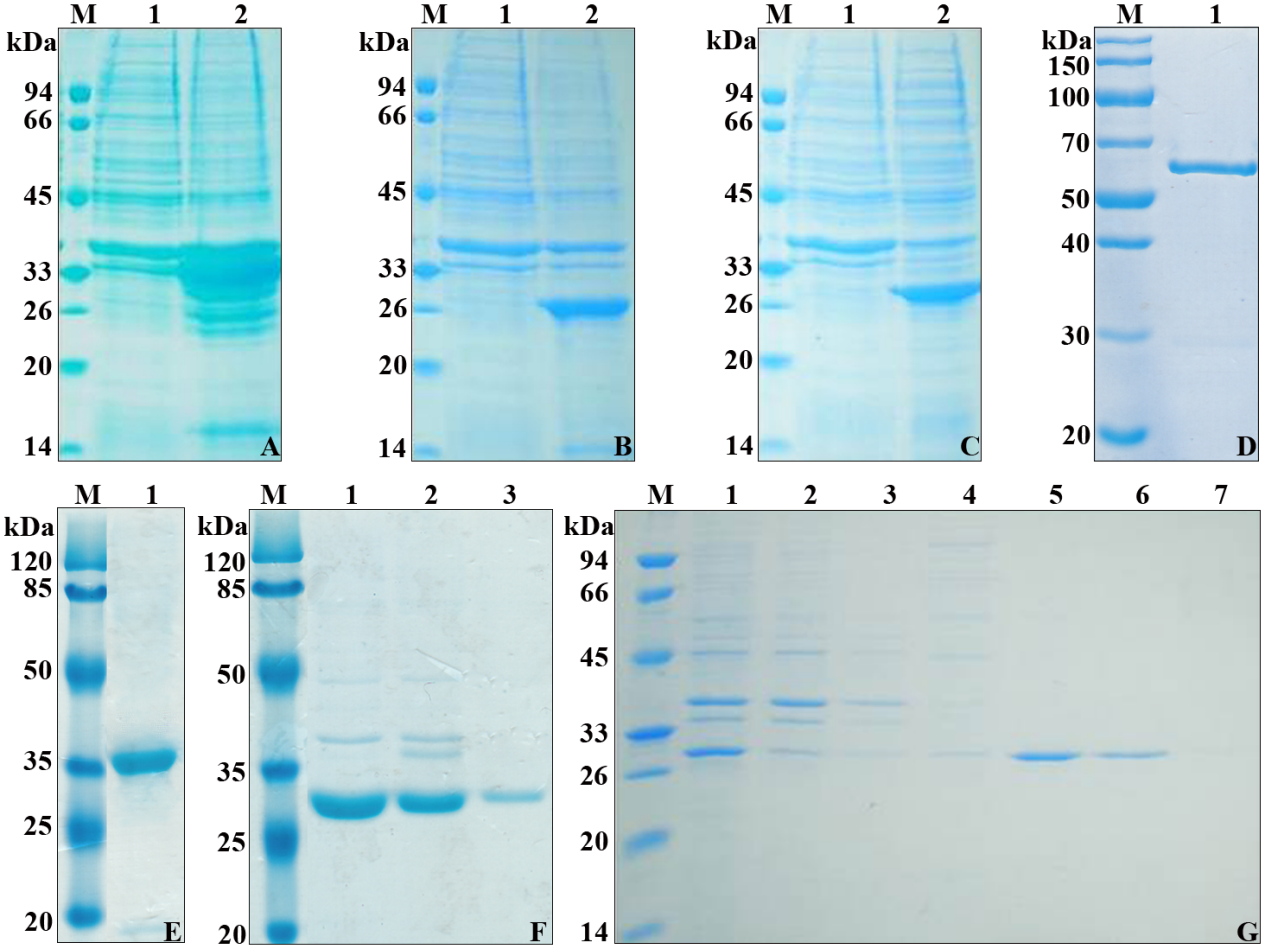
Expression and purification of IE648 and individual domains of Cry1Ie in *E*. *coli* BL21/DE3. A, B and C: Induced expression of domains I, II and III, respectively. Lane M, Protein molecular marker. Lane 1 and 2, supernatant and pellet of bacteria after ultrasonication. Bands of the expected size of 34.6 kDa for domain I, 27.3 kDa for domain II and 27.9 kDa for domain III were present in the pellet fraction. D: SDS-PAGE analysis of purified IE648. Lane M, Protein molecular marker. Lane 1, Purified IE648. E: SDS-PAGE analysis of purified domain I. Lane M, Protein molecular marker. Lane 1, Purified domain I. F: Purification of domain II by Ni-NTA affinity chromatography. Lane M, Protein molecular marker. Lane 1, Inclusion bodies solubilized by using solubilization solutions of Inclusion Body Solubilization and Refolding Kit. Lane 2, Proteins passed through Ni-NTA agarose. Lane 3, Proteins washed by Na_2_CO_3_ buffer (pH 10.2). G: Analysis of purification of domain III by Ni-NTA affinity chromatography. Lane M, Protein molecular marker. Lane 1, Inclusion bodies solubilized by Na_2_CO_3_ (pH 10.2). Lane 2, Proteins passed through Ni-NTA agarose. Lane 3, Proteins washed by Na_2_CO_3_ buffer (pH 10.2). Lane 4~7, Proteins eluted by 20 mM, 50 mM, 100 mM and 150 mM imidazole, respectively.

The three individual domains were cloned into expression vectors using specific primers as described in Materials and Methods. Plasmids harboring these constructions were expressed in *E*. *coli* hosts. The different domains were produced as inclusion bodies, with expected molecular masses of 34.6 kDa for domain I, 27.3 kDa for domain II, and 27.9 kDa for domain III (Fig 1A, 1B and 1C). Different solutions were used to solubilize these domain protein fragments. Fig 1 shows the different Cry1Ie fragments after purification from *E*. *coli* cells. Domain I and domain III were dissolved in alkaline solution of Na_2_CO_3_ as previously reported for domain I [30]. IE648 and domain I was purified as indicated in our previous report [14,30] and the pure samples were shown in Fig 1D and 1E. Domain II protein was dissolved using the solubilization solutions of the Inclusion Body Solubilization and Refolding Kit. A Ni-agarose column was used to purify these protein fragments as described in Materials and Methods. Domain I was eluted with 100 mM imidazole as indicated in our previous report [30], domain II with Na_2_CO_3_ buffer (pH 10.2) (Fig 1F, lane 3), and domain III with 50 mM imidazole (Fig 1G, lanes 5 and 6).

### 3.2. Analysis of binding of IE648 to ACB-PM

**Fig 2:**
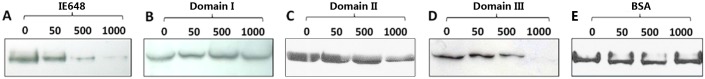
Competition binding assays of IE648 to PM of Asian corn borer. A, Homologous competition binding assays of biotinylated IE648 with different concentrations of unlabeled IE648. The concentration of biotinylated protein is 10 nM, the concentration ratios (molar ratios) between biotinylated protein and unlabeled protein were 1:0, 1:50, 1:500 and 1:1000 (the same below). B, Heterologous competition assays of biotinylated IE648 with different concentrations of unlabeled domain I. C, Heterologous competition assays of biotinylated IE648 with different concentrations of unlabeled domain II. D, Heterologous competition assays of biotinylated IE648 with different concentrations of unlabeled domain III. E, Heterologous competition assays of biotinylated IE648 with different concentrations of unlabeled BSA.

Homologous and heterologous competition binding assays were conducted to determine whether IE648 can bind to PM. The results indicated that IE648 specifically binds to the PM of ACB as shown by the homologous competition binding assay (Fig 2). The heterologous competition assay using the three domains as binding competitors showed that both domain II and III competed with the binding of IE648 to the PM. However, domain III was the more efficient in competing IE648 binding to PM (Fig 2). Domain I showed no competition, similar to the negative control, BSA. These data indicate that domain III plays an important role in IE648 binding with PM of ACB.

**Fig 3:**
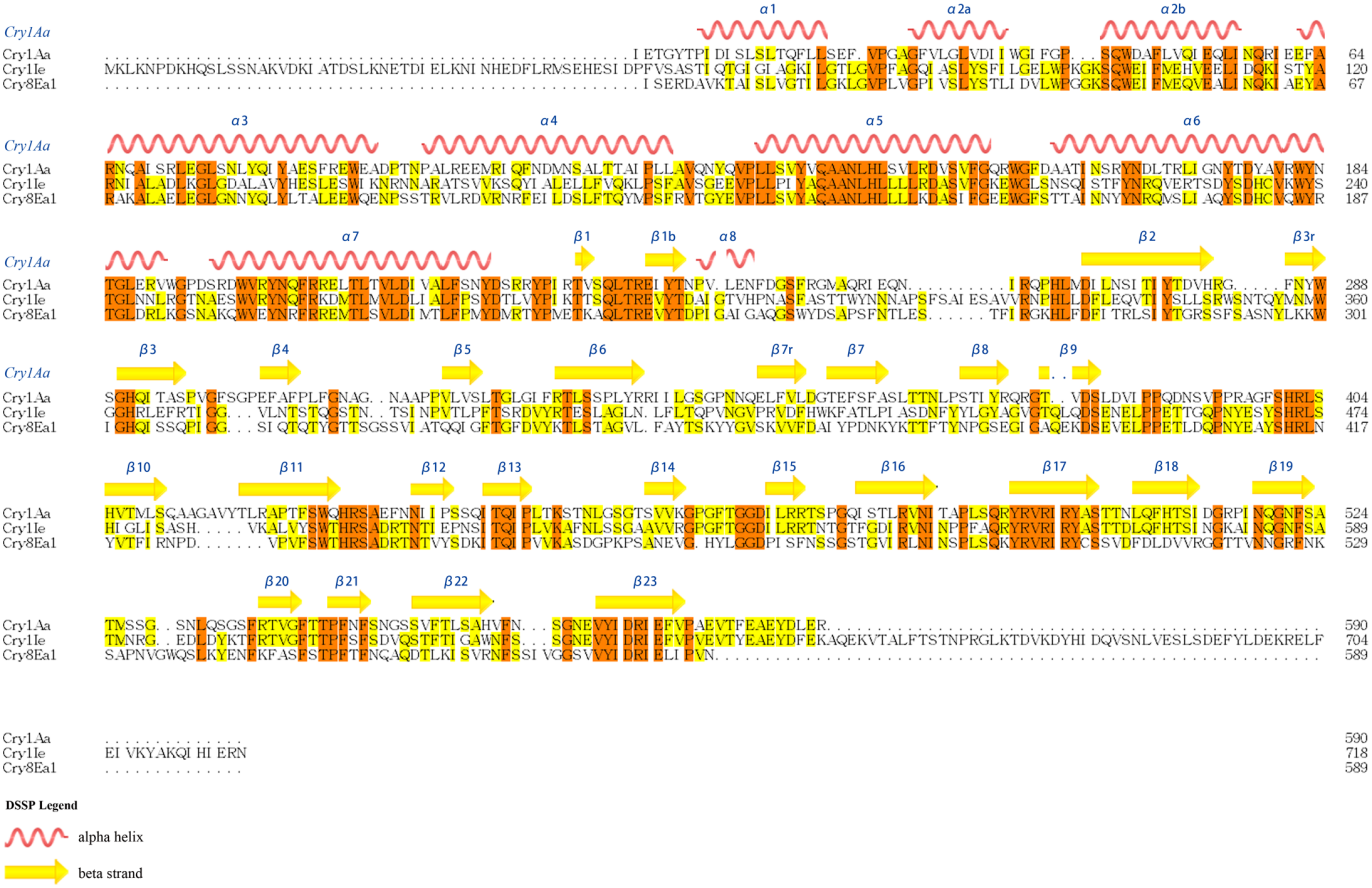
Multiple sequence alignment of primary sequence of Cry1Aa (BAA04468.1), Cry1Ie and Cry8E (AAQ73470.1) proteins. Alignment among three amino acid sequences was achieved through DNAMAN program.

To narrow further the IE648 domain III binding site, several synthetic peptides of domain III were synthesized based on the sequence BLAST and structural model analysis, including conserved peptides and peptides corresponding to the exposed loops. According to a protein BLAST analysis, Cry1Ie toxin has the highest identity with Cry1Aa (34.46% identity) and Cry8E (37.18% identity). Fig 3 shows the alignment of the three sequences.

**Fig 4:**
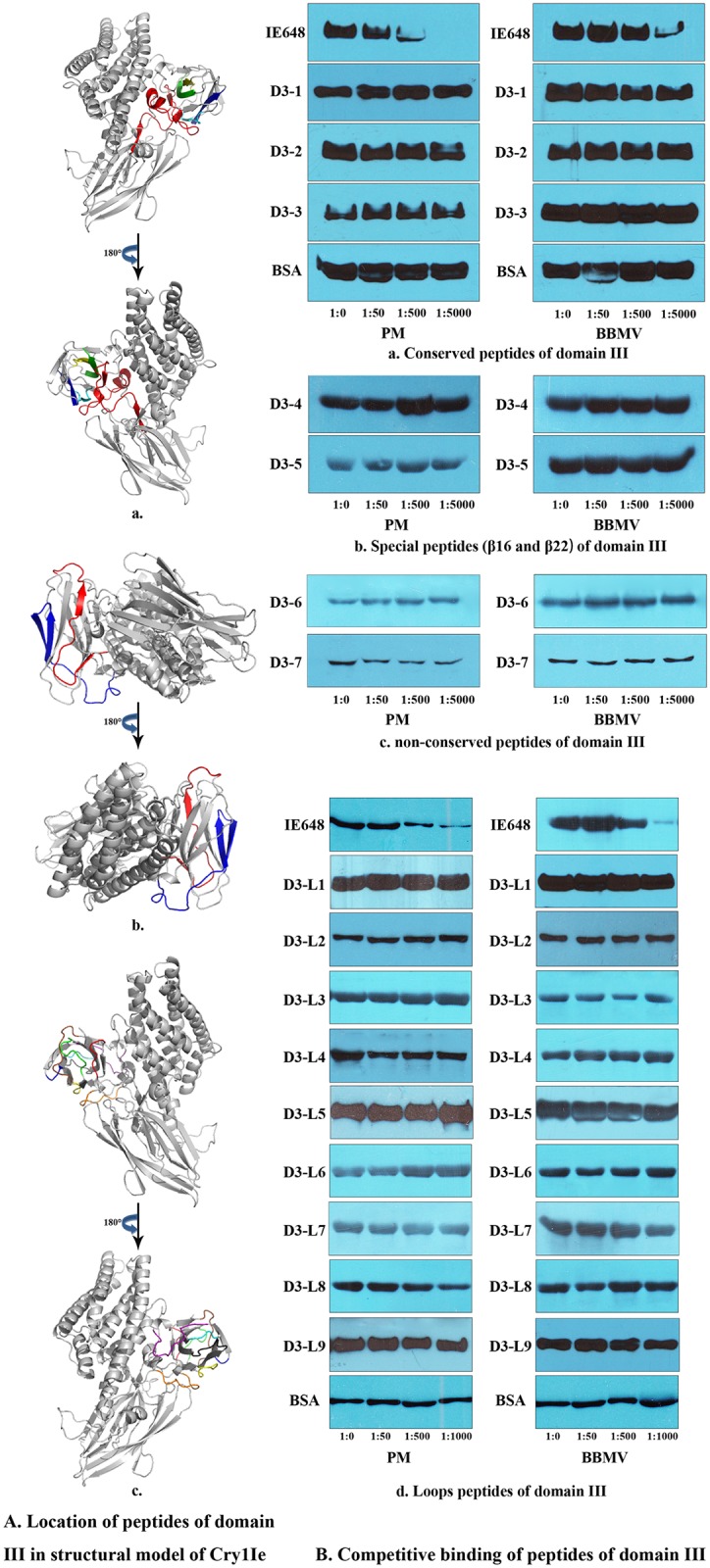
Heterologous competition binding assays of IE648 to PM and BBMV from Asian corn borer with different peptides of domain III. A. Locations of peptides of domain III in the structural model of Cry1Ie. The structural model of Cry1Ie toxin was built using Swiss-Modeling (http://swissmodel.expasy.org/), taking Cry8Ea1 (PDB ID: 3EB7) as a template. (a.) D3-1, red. D3-2, yellow. D3-3, green. D3-4, cyan. D3-5, blue. (b.) D3-6, red. D3-7, blue. (c.) D3-L1, red. D3-L2, orange. D3-L3, yellow. D3-L4, green. D3-L5, cyan. D3-L6, blue. D3-L7, purple. D3-L8, brown. D3-L9, black. B. Competitive binding of peptides of domain III. The concentration of biotin labeled IE648 was 10 nM. Different concentrations of each synthetic peptide were used to compete the binding of labeled IE648 to PM or BBMV, the different molar ratios between biotinylated IE648 and synthetic peptides were 1:0, 1:50, 1:500 and 1:1000.

Three conserved peptides of domain III (Table 2, D3-1, 2, 3) were synthesized. The locations of these peptides in the structural model are shown in Fig 4Aa. The binding competition analysis showed that none of these peptides competed with the binding of IE648 to PM or to BBMV (Fig 4Ba), indicating that these regions are probably not involved in binding with PM or BBMV. It was reported that β16 and β22 of domain III of Cry1Ab toxin play an important role in binding of this toxin to its receptors on the BBMV of *Manduca sexta* [34]. Two specific peptides corresponding to these two regions (Table 2, D3-4, 5) were also synthesized to evaluate their involvement in the interaction with the PM. Their location in the structural model is shown in Fig 4Aa. The binding analysis indicated that β16 and β22 of IE648 were not involved in the interactions with PM or BBMV(Fig 4Bb). Two non-conserved peptides of domain III (Table 2, D3-6, 7) were also investigated. Their location in the structural model is shown in Fig 4Ab. The binding analysis indicated that neither of them were involved in the interactions with PM (Fig 4Bc). According to the analysis of structural model of Cry1Ie, nine peptides located in exposed loops of domain III (Table 2, D3-L1-9) were identified and synthesized, their location is shown in Fig 4Ac. Results of competitive binding assays are shown in Fig 4Bd. Most of the peptides analyzed showed no competition, except for D3-L8 that showed weak competition to PM but not to BBMV (Fig 4Bd).

**Table 2:** Peptides of Cry1Ie domain III.

No.	Position	Secondary Structure	Sequences	Molecular Mass (Da)
D3-1	489–534	β11-β14	YSWTHRSADRTNTIEPNSITQIPLVKAFNLSSGAAVVRGPGFTGGD	4890.45
D3-2	557–567	β17	QRYRVRIRYAS	1467.71
D3-3	633–644	β23	VYIDRIEFVPVE	1478.72
D3-4	544–551	β16	FGDIRVNI	933.40
D3-5	617–625	β22	QSTFTIGAW	1010.30
D3-6	535–556	β15, β16	ILRRTNTGTFGDIRVNINPPFA	2471.34
D3-7	572–598	β18, β19	QFHTSINGKAINQGNFSATMNRGEDLD	2964.38
D3-L1	501–506		TIEPNS	658.20
D3-L2	515–524		AFNLSSGAAV	936.03
D3-L3	539–546		TNTGTFGD	810.30
D3-L4	551–558		INPPFAQR	942.09
D3-L5	568–571		TTDL	449.20
D3-L6	577–579		ING	303.10
D3-L7	587–598		FSATMNRGEDLD	1355.60
D3-L8	607–616		FTTPFSFSDV	1147.60
D3-L9	626–632		NFSSGNE	753.72

Several conserved peptides and peptides located in the loop regions of domain II were also synthesized and analyzed. Quite weak competition of two peptides (D2-L1 and D2-L7) could be observed in the competitive binding of IE648 to PM of ACB, which corroborated the weak binding competition of domain II with IE648 (Fig 2 panel 2C). And two of the conserved peptides were able to compete the binding of IE648 with BBMV (S1 File).

### 3.3. Binding to PM proteins

**Fig 5:**
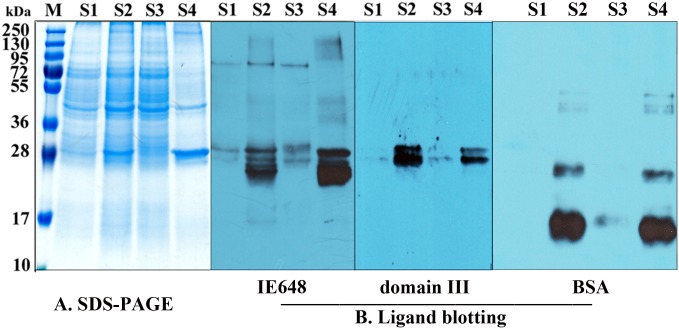
Ligand-blot of IE648 and its domain III with PM of Asian corn borer. A, SDS-PAGE Analysis of the components of PM under different treatments. S1 is the suspension obtained after PM dissolving in 1% Triton X-100. S2 is the homogenate obtained after grinding. S3 is the suspension obtained after the centrifugation of homogenate. S4 is the pellet after the centrifugation. B, Binding of biotinylated IE648, domain III or BSA respectively to components of PM from SDS-PAGE gel.

To determine whether binding of IE648 to PM involves interactions with proteins or chitin from the PM, we first analyzed the binding with PM proteins by ligand-blot assays. Fig 5 shows that IE648 bound to some PM proteins with sizes of 30, 32 and 80 kDa, Fig 5 also shows that two of these proteins with sizes of 30 and 32 kDa were also recognized by domain III. BSA was included as a negative control and found to bind to PM proteins with different size from those bound by IE648 and domain III.

### 3.4. Binding to chitin

**Fig 6:**
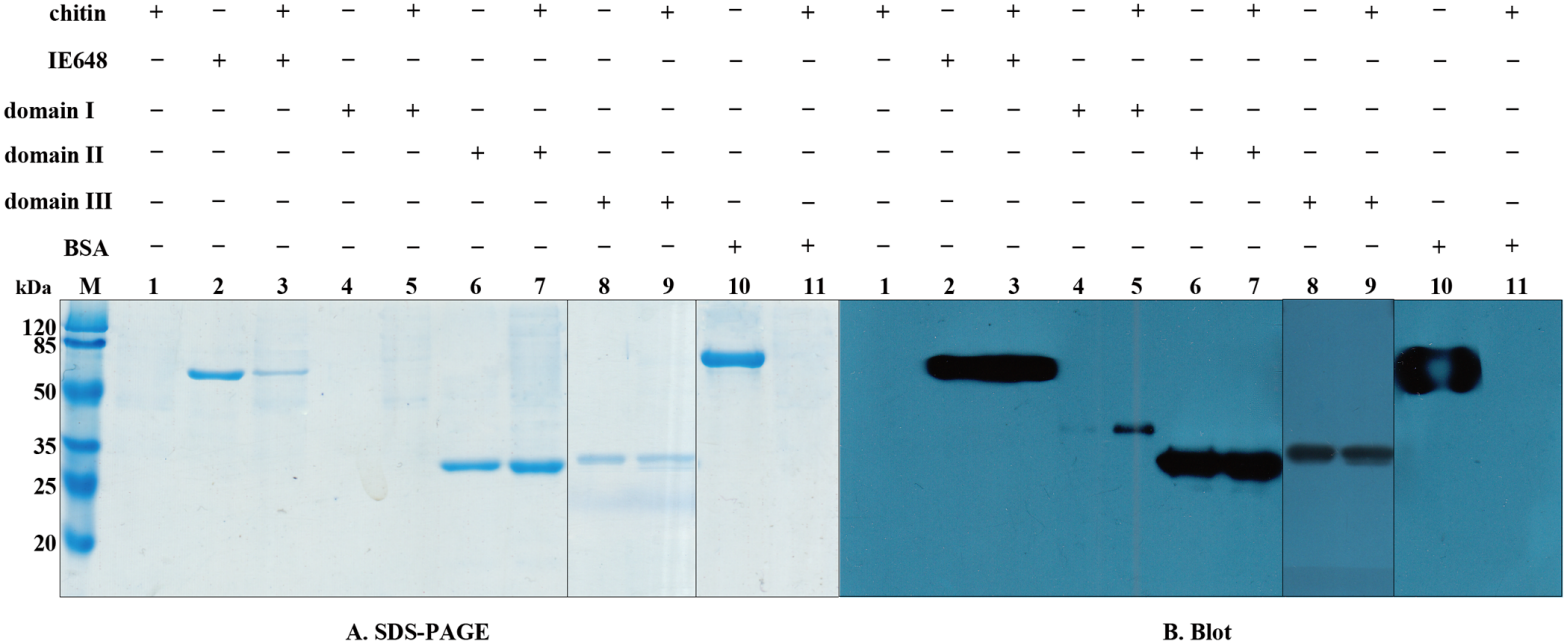
Interaction of chitin with IE648 and its domains. A. SDS-PAGE analysis of different incubation component. B. Blotting result corresponding to each lane of A. Lane 1, chitin. Lanes 2, 4, 6, 8 and 10, positive control of biotinylated proteins in solution directly loaded into the SDS-PAGE: 2, IE648; 4, domain I; 6, domain II; 8, domain III; and 10, BSA. Lanes 3, 5, 7 and 9, binding of biotinylated proteins to chitin analyzed in the pellet after centrifugation: 3, biotinylated IE648 plus chitin; 5, biotinylated domain I plus chitin; 7, biotinylated domain II plus chitin; and 9, biotinylated domain III binding with chitin. Lane 11, biotinylated BSA with chitin, as a negative control. The concentration of biotinylated protein was 10 nM.

To analyze the binding of IE648 with chitin, insoluble chitin was incubated with biotinylated IE648 protein and the bound toxin was separated by centrifugation as described in experimental procedures. The chitin we used in this study is a commercial chitin derived from shrimp. Fig 6 (lane 3) shows that IE648 bound to chitin. However, chitin also bound to domains I, II and III fragments (Fig 6, lanes 5, 7, 9). Fig 6 includes loading controls of biotinylated IE648, domain I, domain II, domain III and BSA proteins, respectively without incubation with chitin (Lanes 2, 4, 6, 8 and 10) as well as BSA, used as a negative control. BSA did not bind to chitin.

## Discussion

Homologous competition binding assays of IE648 to PM from ACB indicated that IE648 binding was specific. Heterologous competition binding assays with the different domains showed that domain II and III competed the binding of IE648 to PM, although domain III fragment was more efficient than domain II in competing binding of IE648 to PM. It is reported that one site of Cry1Ac domain III can recognize the GalNAc on APN receptor [35]. We inferred that domain III might also play a more important role in the binding of Cry1Ie to PM. Different agents that affect PM were used investigated their role as Cry toxin synergist [23], it was reported that Calcofluor although disrupt PM did not enhance insecticidal activity since the Cry1A toxin remains bound to the PM [23]. However other agents such as chitinase enhances insecticidal activity [29]. It has been reported that expression of a modified *cry1Ie* gene in *E*. *coli* and in transgenic tobacco confers resistance to corn borer [36]. The accurate location of the toxin within the insect larvae as well as the binding epitopes would be helpful to access the insecticidal mechanism of Cry1Ie and to improve its toxicity by further mutagenesis of specific regions. More active toxins could also be useful for transgenic application. With the aim of identifying the toxin binding site that participates in the interaction with PM, different peptides corresponding to domain II and III sequences were synthesized, including conserved amino acid regions and predicted exposed loops of Cry1Ie protein. None of the conserved peptides competed the binding of IE648 with the PM from ACB, indicating that conserved regions may not be involved in the interaction with PM. Among the non-conserved peptides and loop-peptides analyzed, peptide D3-L8 (F607-V616), located in an exposed loop region of domain III, competed the binding of IE648 with the PM from ACB, indicating that it is probably involved in the binding interaction with PM of ACB, although the competition was weak. Two loop-peptides of domain II also showed quite weak competition for binding PM. This might be the reason of the weak competition of domain II. However, these regions are different from those involved in binding to BBMV, which means Cry1Ie recognize different receptors on PM and BBMV.

Specific peptides were designed based mainly on their sequence conservation among the Cry toxins, their role in BBMV binding as previously reported and on their structural location. The synthetized peptides covered most of domain III sequence excluding a small 8 residue region (Y599-G606) and 4 amino acid region at the end of domain III (V645-E648). Since no clear binding competition was observed, it is possible that the interaction with PM is conformation dependent and requires 3-dimensional structure to properly position contact points to have significant affinity. It has been reported that domain III of Cry toxins shares a folding structural similarity with different carbohydrate binding domains [35,37]. Thus, it is possible that binding of Cry1Ie to PM proteins could be dependent on carbohydrate binding, which we presume may be structure dependent. This remains to be analyzed. As for the binding to PM proteins, we found that domain III and IE648 bind to two PM proteins with similar molecular weights around 30–32 kDa. We attempted to identify these PM proteins by using Liquid chromatography/ mass spectrometry (LC/MS), but no insect protein matched was found.

PM consists of chitin fibrils and tightly associated proteins. In our result, three domains were found to bind with both chitin and the PM protein, just like the whole IE648 toxin. This binding ability to polysaccharide of Cry1I toxins has never been reported before. Chitin is a polysaccharide composed of β-1,4 linked N-acetylglucosamine (GlcNAc). As discussed above, it was reported that the structure of domain III of Cry1Aa resembles carbohydrate-binding protein domains [37]. Similar bioinformatics analysis of domain III of Cry1Ie was performed (data not shown). The structural model of domain III mainly resembles two proteins, one is *podospora anserina* GH26-CBM35 beta-(1,4)- mannanase (PDB ID 3ZM8, RSM 3.36), the other is carbohydrate binding module of the CBM35 family of Glucuronoxylanase Xyn30D (PDB ID 4QB6, RSM 3.47). They both have molecular function of carbohydrate binding. This indicates that domain III of Cry1Ie have carbohydrate binding potential. Chitin is a long chain polymer of GlcNAc, it is a characteristic component cell walls in fungi and exoskeletons of arthropods such as crustaceans and insects [38]. Valaitis and Podgwaite demonstrated that O-linked glycans present in the glycoconjugates of PM are the target structures for Cry1A binding in Douglas-fir tussock moth larvae since digestion with N-acetyl-alpha-D-galactosaminidase abolished Cry1A toxin-binding to the PM and BBMV components [17]. The stable binding interaction between chitin and toxin proteins in our study also indicates that the monosaccharide residues of chitin, GlcNAc, may play an important role on Cry1I toxin-binding to the PM.

This is the first report that describes that Cry toxins bind PM through domain III. Further work defining the specific amino acids involved in binding to PM by site directed mutagenesis of domain III that affect binding to PM without affecting binding to receptors in BBMV is likely to provide information that could reveal the role of PM binding in Cry toxicity.

## Supporting Information

S1 File Peptides of Cry1Ie domain II **(Table A)**. Heterologous competition binding assays of IE648 to PM and BBMV from Asian corn borer with different peptides from domain II **(Figure A)**.
(RAR)

## References

[1] Ferry N, Edwards MG, Gatehouse JA, Gatehouse AM. Plant-insect interactions: molecular approaches to insect resistance. Curr Opin Biotechnol. 2004; 15(2): 155–161. 15081055 10.1016/j.copbio.2004.01.008

[2] Bravo A, Likitvivatanavong S, Gill SS, Soberon M. *Bacillus thuringiensis*: A story of a successful bioinsecticide. Insect Biochem Mol Biol. 2011; 41(7): 423–431. 10.1016/j.ibmb.2011.02.006 21376122 PMC3689885

[3] Ferre J, Van Rie J. Biochemistry and genetics of insect resistance to *Bacillus thuringiensis* . Annu Rev Entomol. 2002; 47: 501–533. 11729083 10.1146/annurev.ento.47.091201.145234

[4] MacIntosh SC, Stone TB, Jokerst RS, Fuchs RL. Binding of *Bacillus thuringiensis* proteins to a laboratory-selected line of *Heliothis virescens* . Proc Natl Acad Sci U S A. 1991; 88(20): 8930–8933. 1924353 10.1073/pnas.88.20.8930 PMC52624

[5] Tabashnik BE, Finson N, Groeters FR, Moar WJ, Johnson MW, Luo K, et al Reversal of resistance to *Bacillus thuringiensis* in *Plutella xylostella* . Proc Natl Acad Sci U S A. 1994; 91(10): 4120–4124. 8183881 10.1073/pnas.91.10.4120 PMC43736

[6] Hayakawa T, Shitomi Y, Miyamoto K, Hori H. GalNAc pretreatment inhibits trapping of *Bacillus thuringiensis* Cry1Ac on the peritrophic membrane of *Bombyx mori* . FEBS Lett. 2004; 576(3): 331–335. 15498557 10.1016/j.febslet.2004.09.029

[7] Sayyed AH, Gatsi R, Ibiza-Palacios MS, Escriche B, Wright DJ, Crickmore N. Common, but complex, mode of resistance of *Plutella xylostella* to *Bacillus thuringiensis* toxins Cry1Ab and Cry1Ac. Appl Environ Microbiol. 2005; 71(11): 6863–6869. 16269720 10.1128/AEM.71.11.6863-6869.2005 PMC1287713

[8] Song F, Zhang J, Gu A, Wu Y, Han L, He K, et al Identification of *cry1I*-type genes from *Bacillus thuringiensis* strains and characterization of a novel *cry1I*-type gene. Appl Environ Microbiol. 2003; 69(9): 5207–5211. 12957903 10.1128/AEM.69.9.5207-5211.2003 PMC194953

[9] Crickmore N, Zeigler DR, Feitelson J, Schnepf E, Van Rie J, Lereclus D, et al Revision of the nomenclature for the *Bacillus thuringiensis* pesticidal crystal proteins. Microbiol Mol Biol Rev. 1998; 62(3): 807–813. 9729610 10.1128/mmbr.62.3.807-813.1998 PMC98935

[10] Tailor R, Tippett J, Gibb G, Pells S, Pike D, Jordan L, et al Identification and characterization of a novel *Bacillus thuringiensis* delta-endotoxin entomocidal to coleopteran and lepidopteran larvae. Mol Microbiol. 1992; 6(9): 1211–1217. 1588820 10.1111/j.1365-2958.1992.tb01560.x

[11] Gleave AP, Williams R, Hedges RJ. Screening by polymerase chain reaction of *Bacillus thuringiensis* serotypes for the presence of *cryV*-like insecticidal protein genes and characterization of a *cryV* gene cloned from *B*. *thuringiensis subsp*. *kurstaki* . Appl Environ Microbiol. 1993; 59(5): 1683–1687. 8517758 10.1128/aem.59.5.1683-1687.1993 PMC182139

[12] Sekar V, Held B, Tippett J, Amirhusin B, Robeff P, Wang K, et al Biochemical and molecular characterization of the insecticidal fragment of CryV. Appl Environ Microbiol. 1997; 63(7): 2798–2801. 9212427 10.1128/aem.63.7.2798-2801.1997 PMC168576

[13] Wu YE, Zhang J, Cao JP, Gao JG, Huang DF, Song FP. Study on truncated Cry1Ie1 protein from Bacillus thuringiensis. Plant Prot (China). 2003; 29(10): 15–18.

[14] Guo S, Zhang C, Lin X, Zhang Y, He K, Song F, et al Purification of an active fragment of Cry1Ie toxin from *Bacillus thuringiensis* . Protein Expr Purif. 2011; 78(2): 204–208. 10.1016/j.pep.2011.03.006 21421052

[15] Tellam RL, Wijffels G, Willadsen P. Peritrophic matrix proteins. Insect Biochem Mol Biol. 1999; 29(2): 87–101. 10196732 10.1016/s0965-1748(98)00123-4

[16] Zimoch L, Merzendorfer H. Immunolocalization of chitin synthase in the tobacco hornworm. Cell Tissue Res. 2002; 308(2): 287–297. 12037585 10.1007/s00441-002-0546-7

[17] Valaitis AP, Podgwaite JD. *Bacillus thuringiensis* Cry1A toxin-binding glycoconjugates present on the brush border membrane and in the peritrophic membrane of the *Douglas-fir tussock moth* are peritrophins. J Invertebr Pathol. 2013; 112(1): 1–8. 10.1016/j.jip.2012.10.002 23108174

[18] Bolognesi R, Terra WR, Ferreira C. Peritrophic membrane role in enhancing digestive efficiency. Theoretical and experimental models. J Insect Physiol. 2008; 54(10–11): 1413–1422. 10.1016/j.jinsphys.2008.08.002 18761346

[19] Bravo A, Jansens S, Peferoen M. Immunocytochemical localization of *Bacillus thuringiensis* insecticidal crystal proteins in intoxicated insects. J Invertebr Pathol. 1992; 60(3): 237–246.

[20] Denolf P, Jansens S, Peferoen M, Degheele D, Van Rie J. Two Different Bacillus thuringiensis Delta-Endotoxin Receptors in the Midgut Brush Border Membrane of the European Corn Borer, Ostrinia nubilalis (Hubner) (Lepidoptera: Pyralidae). Appl Environ Microbiol. 1993; 59(6): 1828–1837. 16348960 10.1128/aem.59.6.1828-1837.1993 PMC182168

[21] Aranda E, Sanchez J, Peferoen M, Guereca L, Bravo A. Interactions of Bacillus thuringiensis crystal proteins with the midgut epithelial cells of Spodoptera frugiperda (Lepidoptera: Noctuidae). J Invertebr Pathol. 1996; 68(3): 203–212. 8931361 10.1006/jipa.1996.0087

[22] Chen J, Brown MR, Hua G, Adang MJ. Comparison of the localization of *Bacillus thuringiensis* Cry1A delta-endotoxins and their binding proteins in larval midgut of tobacco hornworm, *Manduca sexta* . Cell Tissue Res. 2005; 321(1): 123–129. 15902495 10.1007/s00441-005-1124-6

[23] Rees JS, Jarrett P, Ellar DJ. Peritrophic membrane contribution to Bt Cry delta-endotoxin susceptibility in Lepidoptera and the effect of Calcofluor. J Invertebr Pathol. 2009; 100(3): 139–146. 19320042 10.1016/j.jip.2009.01.002

[24] Adang MJ, Spence KD. Surface morphology of peritrophic membrane formation in the cabbage looper, *Trichoplusia ni* . Cell Tissue Res. 1981; 218(1): 141–147. 7249059 10.1007/BF00210100

[25] Wang P, Granados RR. Molecular structure of the peritrophic membrane (PM): identification of potential PM target sites for insect control. Arch Insect Biochem Physiol. 2001; 47(2): 110–118. 11376457 10.1002/arch.1041

[26] Mohan S, Ma PW, Pechan T, Bassford ER, Williams WP, Luthe DS. Degradation of the *S*. *frugiperda* peritrophic matrix by an inducible maize cysteine protease. J Insect Physiol. 2006; 52(1): 21–28. 16243350 10.1016/j.jinsphys.2005.08.011

[27] Mohan S, Ma PW, Williams WP, Luthe DS. A naturally occurring plant cysteine protease possesses remarkable toxicity against insect pests and synergizes Bacillus thuringiensis toxin. PloS one. 2008; 3(3): e1786 10.1371/journal.pone.0001786 18335057 PMC2262944

[28] Michiels K, Van Damme EJM, Smagghe G. Plant-insect interactions: what can we learn from plant lectins? Arch Insect Biochem Physiol. 2010; 73(4): 193–212. 10.1002/arch.20351 20151457

[29] Liu X, Ma X, Lei C, Xiao Y, Zhang Z, Sun X. Synergistic effects of Cydia pomonella granulovirus GP37 on the infectivity of nucleopolyhedroviruses and the lethality of *Bacillus thuringiensis* . Arch Virol. 2011; 156(10): 1707–1715. 10.1007/s00705-011-1039-3 21643992

[30] Guo SY, Li J, Chen Z, He KL. Penetration of a Single Domain of Bacillus thuringiensis Cry1Ie-Domain I to a Lipid Membrane In vitro. J Integr Agric. 2014; 13(5): 1043–1050.

[31] Jurat-Fuentes JL, Gould FL, Adang MJ. Dual resistance to *Bacillus thuringiensis* Cry1Ac and Cry2Aa toxins in *Heliothis virescens* suggests multiple mechanisms of resistance. Appl Environ Microbiol. 2003; 69(10): 5898–5906. 14532042 10.1128/AEM.69.10.5898-5906.2003 PMC201244

[32] Bradford MM. A rapid and sensitive method for the quantitation of microgram quantities of protein utilizing the principle of protein-dye binding. Anal Biochem. 1976; 72: 248–254. 942051 10.1016/0003-2697(76)90527-3

[33] Padilla C, Pardo-Lopez L, de la Riva G, Gomez I, Sanchez J, Hernandez G, et al Role of tryptophan residues in toxicity of Cry1Ab toxin from *Bacillus thuringiensis* . Appl Environ Microbiol. 2006; 72(1): 901–907. 16391132 10.1128/AEM.72.1.901-907.2006 PMC1352281

[34] Gomez I, Arenas I, Benitez I, Miranda-Rios J, Becerril B, Grande R, et al Specific epitopes of domains II and III of *Bacillus thuringiensis* Cry1Ab toxin involved in the sequential interaction with cadherin and aminopeptidase-N receptors in *Manduca sexta* . J Biol Chem. 2006; 281(45): 34032–34039. 16968705 10.1074/jbc.M604721200

[35] Burton SL, Ellar DJ, Li J, Derbyshire DJ. N-acetylgalactosamine on the putative insect receptor aminopeptidase N is recognised by a site on the domain III lectin-like fold of a *Bacillus thuringiensis* insecticidal toxin. J Mol Biol. 1999; 287(5): 1011–1022. 10222207 10.1006/jmbi.1999.2649

[36] Liu YJ, Song FP, He KL, Yuan Y, Zhang XX, Gao P, et al Expression of a modified *Cry1Ie* gene in *E*. *coli* and in transgenic tobacco confers resistance to corn borer. Acta Biochim Biophys Sin. 2004; 36(4): 309–313. 15253158 10.1093/abbs/36.4.309

[37] de Maagd RA, Bravo A, Berry C, Crickmore N, Schnepf HE. Structure, diversity, and evolution of protein toxins from spore-forming entomopathogenic bacteria. Annu Rev Genet. 2003; 37: 409–433. 14616068 10.1146/annurev.genet.37.110801.143042

[38] Horst MN. The biosynthesis of crustacean chitin. Isolation and characterization of polyprenol-linked intermediates from brine shrimp microsomes. Arch. Biochem. Biophys. 1983; 223: 254–263 6859859 10.1016/0003-9861(83)90590-8

